# Proteomic data of seminal plasma and spermatozoa of four purebred dogs

**DOI:** 10.1016/j.dib.2020.105498

**Published:** 2020-04-06

**Authors:** Michelle Silva Araujo, Otávio Luís de Oliveira Henriques Paulo, Cristiane Sella Paranzini, Caroline Scott, Viviane Maria Codognoto, Camila de Paula Freitas Dell'Aqua, Frederico Ozanam Papa, Fabiana Ferreira de Souza

**Affiliations:** São Paulo State University, Botucatu, São Paulo, Brazil

**Keywords:** Proteomic, Mass spectrometry, Purebred dog, Semen

## Abstract

Semen contains several proteins that are important to fertilization and to identify reproductive failures. There are proteins that are specie-specific expressed, although differs among several breeds. This article provides experimental data describing the protein profile of seminal plasma and spermatozoa of four healthy purebred dogs: Golden Retriever (*n*=3), Bernese Mountain Dog (*n*=4), Great Dane (*n*=3), and Maremmano-Abruzzese Sheepdog (*n*=3), housed at São Paulo state, Brazil. Semen samples were collected by manual stimulation of the penis in a presence of a teaser bitch, when possible. The seminal plasma and sperm cells were separated by centrifugation and prepared for mass spectrometry. The gene ontology annotation of the proteins found is described. This is the first time that proteomic profile of the semen of purebred dogs is described. These data are a valuable resource to improve the biotechnologies of reproduction applied to canid species.

Specifications table

**Table:** 

Subject	Veterinary Medicine
Specific subject area	Canine reproduction
Type of data	Text
How data were acquired	Instruments: Q-Tof mass spectrometer (Micromass^Ⓡ^ Q-Tof PREMIER Mass Spectrometer, Waters Corporation, Milford, MA, USA) Model and make of the instruments used: Spectra were acquired using the MassLynx™ software v.4.1. (Waters Corporation, Mildford, MA, USA). The gene ontology annotation of the proteins found within the samples was obtained using the UniprotKB website (www.uniprot.org), and considered the molecular function, biological process, cellular component and protein class categories.
Data format	Raw data
Parameters for data collection	Healthy purebred dogs (Golden Retriever, Bernese Mountain Dog, Maremmano-Abruzzese Sheepdog, Great Dane), from different kennels located at São Paulo state, Brazil. Only ejaculated with semen characteristics considered within the normal range for dogs, were used for these data.
Description of data collection	Entire second fraction and a portion of the third semen fraction were collected into a silicone funnel attached to a graduated plastic tube by manual stimulation of the penis in the presence of a teaser bitch, when possible. The seminal plasma and sperm cells were separated by centrifugation. All the samples were prepared for mass spectrometry.
Data source location	City: Cotia and Botucatu (Bernese Mountain Dog), Sorocaba (Great Dane and Golden Retriever) and Itapira (Maremmano-Abruzzese Sheepdog) Country: Brazil Latitude and longitude (and GPS coordinates) for collected samples/data: Cotia: Latitude 23^o^ 36’ 15’’ south, Longitude 46^o^ 55’ 27’’ west Botucatu: Latitude 22^o^ 53’ 25’’ south, Longitude: 48^o^ 27’ 19’’ west Sorocaba: Latitude 23^o^ 30’ 06’’ south, Longitude 47^o^ 27’ 29’’ west Itapira: Latitude 22^o^ 26’ 11’ south, Longitude 46^o^ 49’ 20’’ west
Data accessibility	In a public repository Repository name: Mendeley Data Data identification number: doi:10.17632/739k25yj4s.4 Direct URL to data: (https://data.mendeley.com/datasets/739k25yj4s/4)

## Value of the data

• This is the first time a comparative protein expression profile of the proteins found in the spermatozoa and seminal plasma of purebred dogs is described, which are very important to improve biotechnologies of reproduction in canid species and also in human.
• Differences in spermatozoa and seminal plasma proteomic profile were observed among breeds.
• These data can be useful for researchers, pharmaceutical industry and veterinarians.
• These data can be used to compare additional findings about proteomic profile of semen from another species.

## 1 Data description

The four datasets supporting this article are available in the Mendeley Data online repository (https://data.mendeley.com/datasets/739k25yj4s/4) which corresponds to all the sperm cell or seminal plasma proteins found and their respectively gene ontology. Each dataset corresponding to sperm cell and seminal plasma proteins contains files per dog by breed as:

Sperm cell proteins = Contains all files (.dat) for each dog with all sperm cell proteins found. The files of each dog are described by the name of the breed and numbered per dog:

• Golden Dog 1S.dat = This file contains all the seminal plasma proteins found for one Golden Retriever dog breed.
• Bernese Dog 1S.dat = This file contains all the seminal plasma proteins found for one Bernese Mountain Dog dog breed.
• Great Dane Dog 1S.dat = This file contains all the seminal plasma proteins found for one Great Dane dog breed.
• Maremmano Dog 1S.dat = This file contains all the seminal plasma proteins found for one Maremmano-Abruzzese Sheepdog.

Seminal Plasma proteins = Contains all files (.dat) for each dog with all sperm cell proteins found. The files of each dog are described by the name of the breed and numbered per dog:

• Golden Dog 1.dat = This file contains all the seminal plasma proteins found for one Golden Retriever dog breed.
• Bernese Dog 1.dat = This file contains all the seminal plasma proteins found for one Bernese Mountain Dog dog breed.
• Great Dane Dog 1.dat = This file contains all the seminal plasma proteins found for one Great Dane dog breed.
• Maremmano Dog 1.dat = This file contains all the seminal plasma proteins found for one Maremmano-Abruzzese Sheepdog.

The dataset corresponding to gene ontology of sperm cell and seminal plasma proteins contain three files:

• Table S1 = This file contains all the proteins found in seminal plasma of evaluated dogs and their respective gene ontology.
• Table S2 = This file contains all the proteins found in spermatozoa of all dogs evaluated and their respective gene ontology.
• Table S3 = This file contains common proteins found in seminal plasma and spermatozoa of evaluated dogs and their respective gene ontology.

## 2 Experimental design, materials and methods

### 2.1 Ethical aspects

The collection of data was approved by the Ethical Committee for the Use of Animals in Research of the FMVZ-UNESP, with the permit number: 0007/2017.

### 2.2 Reagents

All reagents used were of high purity and purchased from Sigma-Aldrich (St. Louis, MO, USA), Waters Corp. (Barueri, São Paulo, Brazil), GE Healthcare Life Sciences (São Paulo, São Paulo, Brazil), Merck S.A. (São Paulo, São Paulo, Brazil) and Thermo Fisher Scientific (São Paulo, São Paulo, Brazil), otherwise will be stated.

### 2.3 Evaluation of the dogs

Prior semen collection, anamnesis, clinical and reproductive evaluation of 13 purebred dogs [Golden Retriever (*n* = 3), Bernese Mountain Dog (*n* = 4), Great Dane (*n* = 3), and Maremmano-Abruzzese Sheepdog (*n* = 3)], from different kennels located at São Paulo state, were performed to assess their health condition and to discard any reproductive pathologies. The first ejaculate of all dogs was discarded to avoid obtaining old sperm cells that might contain more morphological defects. The ages of each dog according to each attached file are described below:

• Golden Dog 1 = 3 years
• Golden Dog 2 = 4 years
• Golden Dog 3 = 4 years and 11 months
• Bernese Dog 1 = 3 years
• Bernese Dog 2 = 2 years and 6 months
• Bernese Dog 3 = 1 year and 1 month
• Bernese Dog 4 = 1 year
• Great Dane Dog 1 = 2 years
• Great Dane Dog 2 = 1 year and 2 months
• Great Dane Dog 3 = 1 year and 2 months
• Maremmano Dog 1 = 4 years and 2 months
• Maremmano Dog 2 = 3 years and 3 months
• Maremmano Dog 3 = 4 years and 7 months

### 2.4 Semen collection

The entire second and a portion of the third semen fraction were collected using a silicone funnel attached to a graduated plastic tube by manual stimulation of the penis in the presence of a teaser bitch, when possible.

### 2.5 Semen quality assessment

In the kennel, an aliquot 10 µL of semen were subjectively evaluated on pre-warmed glass slide (37° C) covered with a glass coverslip under light microscope (100X magnification) for motility (0-100%) and vigor (0-5). Sperm morphology and vitality were evaluated with a semen smear (5 µL) stained with eosin/nigrosine (5 µL) (BotuVital^Ⓡ^, Botupharma, Botucatu, Brazil) and observed 200 spermatozoa under a light microscope at 1,000X magnification, in which the spermatozoa stained in red were considered dead, and those not stained as live. Sperm morphology was classified in major and minor defects according to Blom and Christensen (1972) [2]. The sperm concentration was accessed by a Neubauer chamber after semen dilution of 1:100 in formol-saline [3]. Only ejaculated with seminal parameters considered within the normal range for dogs, according to Kustritz (2007) [1], were considered for these data.

### 2.6 Seminal plasma and spermatozoa proteins extraction

After semen collection, seminal plasma and sperm cells were separated by centrifugation at 800g for 10 min. Then, seminal plasma (supernatant) was chilled at 5°C in a foam box (Botuflex^Ⓡ^, Botupharma, Botucatu, São Paulo, Brazil). Spermatozoa (pellet) was washed three times with a buffer (50 mmol TRIS pH 7.2) containing protease inhibitors (0.8 mmol EDTA, 1.0 µg/mL aprotinin, 1.0 µg/mL leupeptin e 35.0 µg/mL phenylmethylsulfonil fluoride - PMSF); and then chilled at 5° C as previously reported for seminal plasma. Both samples were transported to the laboratory of proteomics (maximum time between sampling and arrival in the laboratory was 3 h) located at the Department of Animal Reproduction and Veterinary Radiology of São Paulo State University, Botucatu Campus, Botucatu, Brazil. At the laboratory, all the samples were maintained at -20 °C until protein extraction.

Seminal plasma and spermatozoa samples were thawed at ice bath. Seminal plasma samples were recentrifuged at 10,000g for 30 min at 4°C to remove any remaining sperm and cellular debris, and the supernatant was recovered for protein extraction. A protein solubilization buffer (150 mM NaCl 1% Triton X-100, 1% sodium deoxycholate, 0.1% SDS) [4] was added to spermatozoa samples at a final concentration of 200 × 10^6^ spermatozoa/mL.

Spermatozoa were sonicated according to Baker et al. (2010) [5] and modified by Souza et al. (2009) [6]. A 3.0 mm probe was used and 20% amplitude for 30 s, in ice bath, performed in 10 series, with 1 min arrest between series. After sonication, the samples were centrifuged at 10,000 g at 4°C for 30 min.

Total proteins concentration was determined in duplicate by A280 method spectrophotometer (NanoDrop A280, Thermo Scientific NanoDrop One Microvolume UV-VIS Spectrophotometers, Waltham, Massachusetts, EUA).

In-solution digestion of seminal plasma proteins was performed according to Codognoto et al (2018) [7].

### 2.7 SDS-PAGE assay

This assay was also performed at the same laboratory of proteomics that seminal samples were stored. SDS-PAGE was used to remove extraction buffer components from spermatozoa samples which may compromise the nanochromatography column. A total of 50 µg protein was used in 12% separation gel at a vertical mini gel (Hoefer MiniVE Vertical Electrophoresis System, GE HealthCare, São Paulo, SP, Brazil). The run was stopped as the sample reached the separation gel, in which a unique band was formed. Then, the gel was fixed in a solution containing 40% (v/v) ethanol and 10% (v/v) acetic acid for 60 min and stained with colloidal coomassie blue [8], [9], [10]. Gel was maintained under distilled water until background whitening and then stained protein band was cut off. Bands were distained with 50% (v/v) methanol, 2.5% (v/v) acetic acid in ultra-pure water for 1 h, at room temperature. Solution was removed and added again. This procedure was repeated for more three times. Distain solution was removed, and acetonitrile 100% (v/v) was added for 5 min. Acetonitrile was removed, and this procedure was repeated once. Remaining acetonitrile was evaporated in a vacuum concentrator for 2 to 3 min. Then, 10 mM DTT in 100 mM ammonium bicarbonate were added and incubate for 30 min at room temperature. A fast spin was performed to remove DTT solution and 50 mM IAA in 100 mM ammonium bicarbonate was added and protected from light for 30 min. A fast spin was performed to remove IAA solution.

Gel fragments were washed with 100 mM ammonium bicarbonate for 10 min and the solution was removed. Gel dehydration was done with acetonitrile 100% (v/v), incubated for 5 min at room temperature and removed. The gels were rehydrated with100 mM ammonium bicarbonate for 10 min. A fast spin was done to remove this solution and the gels were dehydrated again with acetonitrile as previously described for two more times. Remaining solution was evaporated in a vacuum concentrator for 2 to 3 min and 30 to 50 µL of trypsin solution (20 µL trypsin in 1,000 µL of cold 50 mM ammonium bicarbonate, at a final concentration of 20 ng/µL of trypsin) was added and the gels were rehydrated in an ice bath for 30 min. A fast spin was performed to remove the exceed trypsin solution and 5 to 20 µL of 50 mM ammonium bicarbonate were added until cover the gels, at 37°C, overnight. Then, 10 µL of 5% formic acid (v/v) in ultra-pure water was added and incubated at room temperature for 10 min, make a fast spin and save the supernatant in another tube. A 12 µL of 5% formic acid (v/v) in 5% acetonitrile (v/v) were added until cover the gels, incubated for 10 min at room temperature. A fast spin was done, and the supernatant was deposited at the same tube contained the previously supernatant. This last step was repeated. The sample was dried until obtain approximately 1 µL and storage at -20°C for mass spectrometry.

### 2.8 Mass spectrometry

For mass spectrometry, the samples were thawed, diluted in 0.1% formic acid in the proportion of 0.7 µg protein/µL, homogenized in a tube shaker and centrifuged at 1,100g for 5 min. Next, 20 µL of the supernatant was deposited in specific tubes for analysis in the mass spectrometer (Clear glass 12 × 32 mm screw neck total recovery vial with lid, Waters Corporation, Milford, MA, USA) [7].

For protein analysis by mass spectrometry, 4.5 µL aliquot resulting from peptide digestion was separated by a C18 (100 µm x 100 mm) RP-nano UPLC column (Waters nanoACQUITY UPLC, Waters Corporation, Milford, MA, USA) coupled to the Q-Tof mass spectrometer (Micromass^Ⓡ^ Q-Tof PREMIER Mass Spectrometer, Waters Corporation, Milford, MA, USA) with a nanoelectrospray source at a flow rate of 0.600 µL/min. The samples were evaluated in duplicate. A gradient of 2 to 90% acetonitrile in 0.1% formic acid was maintained for 45 min. The voltage of the nanoelectrospray was maintained at 3.5 kV, cone voltage of 30 V and source temperature of 100 µC. The instrument was operated in top three mode in which a mass spectrum (MS) is acquired followed by MS/MS of the three most intense peaks detected. After MS/MS fragmentation, the ion was maintained in the exclusion list for 60 s. For endogenous cleavage peptides analysis, a real exclusion time was used.

Spectra were acquired using the MassLynx™ software v.4.1 (Waters Corporation, Milford, MA, USA) and the raw data files were converted to a peak list format (.mgf, mascot generic format) without adding the scans and searched against UniprotSProt_012015 (http://www.uniprot.org/) *Mammalia* taxonomy database, using the Mascot tool 2.3.02 *version* and Mascot Distiller MDRO 2.4.0.0 *version* (Matrix Science Inc, Boston, MA, USA). Relative quantification of each protein in the mixture was determined by exponentially modified protein abundance index (emPAI), obtained from Mascot Distiller software [11].

Search parameters included trypsin as protease, with a maximum of 1 cleavage lost; carbamidomethylation of cysteine as fixed modification and methionine oxidation as a variable modification. Tolerance of 0.1 Da for both precursor (MS) and fragment (MS/MS) of ions and monoisotopic molecular mass was used.

### 2.9 Gene ontology assessment

The gene ontology annotation of the proteins found was obtained using the UniprotKB website (www.uniprot.org) [12], and considered the molecular function, biological process and cellular component categories. Figures on gene ontology were assembled using the online software *Panther* (version 10) (http://www.pantherdb.org) [13].

## Appendix Supplementary materials

Image, application 1

## References

[1] Kustritz M.V.R. (2007). The value of canine semen evaluation for practitioners. Theriogenology.

[2] Blom E., Christensen N.O.systematic search for abnormalities in testis-epididymis in pedigree bulls in Denmark. studies on pathological conditions in the testis, epididymis, and accessory sex glands in the bull. VII. Denmark Vet landbohojsk Arsskr. 1972

[3] Freshman J.L. (2002). Semen collection and evaluation. Clin. Tech. Small Anim. Pract..

[4] Peach M., Marsh N., MacPhee D.J. (2012). Protein solubilization: attend to the choice of lysis buffer. Protein Electrophoresis.

[5] Baker M.A., Reeves G., Hetherington L., Aitken R.J. (2010). Analysis of proteomic changes associated with sperm capacitation through the combined use of IPG-strip pre-fractionation followed by RP chromatography LC-MS/MS analysis. Proteomics.

[6] Souza F.F.D., Chirinea V.H., Martins M.I.M., Lopes M.D. (2009). Osteopontin in seminal plasma and sperm membrane of dogs. Reprod. Domest. Anim..

[7] Codognoto V.M., Yamada P.H., Schmith R.A. (2018). Functional insights into the role of seminal plasma proteins on sperm motility of buffalo. Anim. Reprod. Sci..

[8] Luo C., Yang X., Fu Q. (2006). Picoliter‐volume aqueous droplets in oil: electrochemical detection and yeast cell electroporation. Electrophoresis.

[9] Neuhoff V., Stamm R., Eibl H. (1985). Clear background and highly sensitive protein staining with Coomassie Blue dyes in polyacrylamide gels: a systematic analysis. Electrophoresis.

[10] Neuhoff V., Arold N., Taube D., Ehrhardt W. (1988). Improved staining of proteins in polyacrylamide gels including isoelectric focusing gels with clear background at nanogram sensitivity using Coomassie Brilliant Blue G‐250 and R‐250. Electrophoresis.

[11] Ishihama Y., Oda Y., Tabata T. (2005). Exponentially modified protein abundance index (emPAI) for estimation of absolute protein amount in proteomics by the number of sequenced peptides per protein. Mol. Cell. Proteom..

[12] Boutet E., Lieberherr D., Tognolli M. (2016). UniProtKB/Swiss-Prot, the manually annotated section of the UniProt KnowledgeBase: how to use the entry view. Plant Bioinformatics.

[13] Mi H., Poudel S., Muruganujan A., Casagrande J.T., Thomas P.D. (2016). PANTHER version 10: expanded protein families and functions, and analysis tools. Nucl. Acids Res..

